# Increased Stathmin1 Expression in the Dentate Gyrus of Mice Causes Abnormal Axonal Arborizations

**DOI:** 10.1371/journal.pone.0008596

**Published:** 2010-01-06

**Authors:** Kohei Yamada, Shinsuke Matsuzaki, Tsuyoshi Hattori, Ryusuke Kuwahara, Manabu Taniguchi, Hitoshi Hashimoto, Norihito Shintani, Akemichi Baba, Natsuko Kumamoto, Kazuo Yamada, Takeo Yoshikawa, Taiichi Katayama, Masaya Tohyama

**Affiliations:** 1 Department of Anatomy and Neuroscience, Graduate School of Medicine, Osaka University, Suita, Osaka, Japan; 2 United Graduate School of Child Development, Osaka University, Kanazawa University and Hamamatsu University School of Medicine, Suita, Osaka, Japan; 3 Department of Molecular Neuropsychiatry, Graduate School of Medicine, Osaka University, Suita, Osaka, Japan; 4 Laboratory of Molecular Neuropharmacology, Graduate School of Pharmaceutical Sciences, Osaka University, Suita, Osaka, Japan; 5 Laboratory of Molecular Psychiatry, RIKEN Brain Science Institute, Wako, Saitama, Japan; RIKEN Brain Science Institution, Japan

## Abstract

Pituitary adenylate cyclase-activating polypeptide (PACAP) is involved in multiple brain functions. To clarify the cause of abnormal behavior in PACAP deficient-mice, we attempted the identification of genes whose expression was altered in the dentate gyrus of PACAP-deficient mice using the differential display method. Expression of stathmin1 was up-regulated in the dentate gyrus at both the mRNA and protein levels. PACAP stimulation inhibited stathmin1 expression in PC12 cells, while increased stathmin1expression in neurons of the subgranular zone and in primary cultured hippocampal neurons induced abnormal arborization of axons. We also investigated the pathways involved in PACAP deficiency. Ascl1 binds to E10 box of the stathmin1 promoter and increases stathmin1 expression. Inhibitory bHLH proteins (Hes1 and Id3) were rapidly up-regulated by PACAP stimulation, and Hes1 could suppress Ascl1 expression and Id3 could inhibit Ascl1 signaling. We also detected an increase of stathmin1 expression in the brains of schizophrenic patients. These results suggest that up-regulation of stathmin1 in the dentate gyrus, secondary to PACAP deficiency, may create abnormal neuronal circuits that cause abnormal behavior.

## Introduction

PACAP is a neuropeptide that is expressed in the brain as well as in the neurons of a number of peripheral organs and it is involved in various neurobiological functions, such as neurotransmission and neural plasticity [1], [2]. It also has a neurotrophic effect via three heptahelical G protein-coupled receptors, one of which is specific for PACAP (PAC_1_ receptor) and two others that are shared with vasoactive intestinal polypeptide (VPAC_1_ and VPAC_2_ receptors) [3]. Recently, mice that lack *Adcyap1*, the gene encoding PACAP, (*Adcyap1^−/−^* mice) were developed [2], [4]. *Adcyap1^−/−^* mice display remarkable behavioral abnormalities providing evidence that PACAP plays a previously uncharacterized role in the regulation of psychomotor behavior. When placed into a novel environment, such as an open field, the mutants display significantly increased locomotor activity with minimal time spent habituating themselves to the environment, and less time engaged in licking and grooming behavior. The mutants also show explosive jumping behavior in the open field and increased exploratory behavior [2], [5]. These behavioral abnormalities may be due to perturbation of monoamine neurotransmission because serotonin metabolite 5-hydroxyindoleacetic acid is slightly decreased in the cerebral cortex and striatum of PACAP-deficient mice, and hyperactive behavior is ameliorated by the antipsychotic drug, haloperidol [2]. In addition, the jumping behavior is suppressed by drugs that elevate extracellular serotonin, such as the selective serotonin reuptake inhibitors [6]. *Adcyap1^−/−^* mice also showed increased immobility in a forced swimming test, which was reduced by the antidepressant, desipramine [7]. In addition it is known that PAC_1_-deficient mice exhibit reduced social behavior [8]. And, it is also known that PAC_1_-deficient mice exhibit increased fear conditioning and a reduction of LTP [9]. A previous association study reported that several single nucleotide polymorphisms (SNPs) in the vicinity of the PACAP gene locus were associated with schizophrenia [10]. However, none of genome wide association studies showed association of this gene with schizophrenia [11]. *Disrupted-In-Schizophrenia 1* (*DISC1*) has been identified as a potential susceptibility gene for major psychiatric disorders [12], [13]. We previously identified several DISC1-interacting factors [14], [15], [16]. DISC1-binding zinc finger protein (DBZ) is one of these factors. We found that PACAP up-regulates DISC1 expression and markedly reduced the association of DISC1 with DBZ in PC12 cells, and that a DISC1-binding domain of DBZ reduces neurite length in PC12 cells following PACAP stimulation in primary cultured hippocampal neurons [12]. Therefore, these results suggest that PACAP may play a role in mental disorders such as schizophrenia.

However, nothing is known about the mechanisms of PACAP deficiency-induced psychiatric illness, so this study was performed to investigate these mechanisms.

The localization of PAC_1_ mRNA in the neurons of rat and mice brains has been examined by *in situ* hybridization [17]. Neurons showing intense signals for PAC_1_ mRNA were found in the dentate gyrus of the hippocampus, olfactory bulb, second layer of the cerebral cortex, and several hypothalamic areas. In addition, an atrophy of the hippocampus had been reported in schizophrenic patients [10]. To elucidate molecular events associated with PACAP deficiency in the mouse brain that could be relevant to schizophrenia, therefore we attempted to detect PACAP deficiency-regulated genes in the dentate gyrus using the differential display (DD) method and found that stathmin1 expression was up-regulated in the dentate gyrus of PACAP-deficient mice. Stathmin1 is expressed at high levels by neurons and glial cells of the brain [18], [19]. It interacts with tubulin and destabilizes microtubules [20]. However, the detailed functions and regulatory mechanisms of stathmin1 in the formation of neural networks are unclear.

In this study, we found that an increase of stathmin1 expression induced abnormal sprouting of neurons in the dentate gyrus, and we showed that stathmin1 was regulated by a basic helix loop helix (bHLH) factor via a PACAP-dependent molecular signaling pathway.

## Methods

### Animals

*Adcyap^−/−^* mice and wild-type littermates were provided by the Baba lab, Laboratory of Molecular Neuropharmacology, Graduate School of Pharmaceutical Sciences, Osaka University. Pregnant female rats were deeply anesthetized with sodium pentobarbital. Brains were dissected from day 18 embryos and cultured. All animal experiments were carried out in accordance with a protocol approved by the Institutional Animal Care and Use Committee of Osaka University.

### Construction of the stathmin1, Ascl1, Hes1 and Id3 expression vectors

Mouse stathmin1 cDNA was subcloned into pEGFP-C3 (Clontech) to generate stathmin1 with green fluorescent protein (GFP) fused to the N-terminal (GFP-stathmin1) and into a bicistronic expression vector (pIRES2-EGFP; Clontech) to produce stathmin1–IRES–GFP. A mouse Hes1 expression plasmid, pCI-Hes1, and Hes1 antibody were kind gifts from Prof. R. Kageyama. Rat Ascl1 and Id3 were sub-cloned into pCI-neo (Clontech).

### Cell culture and transfection

PC12 cells were cultured in Dulbecco's modified Eagle's medium (DMEM) containing 10% horse serum and 5% FBS in a 5% CO_2_/95% air humidified atmosphere at 37°C. Twenty four hours after plating, PACAP was applied. The inhibitors were added 1 h before PACAP treatment. Rat primary hippocampal neurons were prepared from day 18 embryos using nerve cell culture system MB-X9901 (Sumitomo Bakelite, Tokyo, Japan) as described in the Sumilon Protocol N-4.2. Neurons were plated on poly-L-lysine coated chamber slides and cultured in MEM with 5% FCS in a 5% CO_2_/95% air humidified atmosphere at 37°C. Approximately 1×10^5^ neurons or 6×10^5^ PC12 cells were transfected with 2 µg or 1 µg of pGFP-Stathmin1, pStathmin1–IRES–GFP or a GFP only expression vector using Lipofectamine 2000 (Invitrogen, Carlsbad, CA, USA).

### Construction of rat stathmin1 promoter-luciferase plasmids

Rat stathmin1 genomic sequence (PubMed accession no. NC_005104) was identified using a BLAST search with stathmin1 cDNA sequence, and a 1.9 kb region corresponding −1534 to +325 was amplified by PCR using forward primer 5′- and reverse primer 5′- containing KpnI and NheI sites and subcloned into the KpnI-NheI site of the pGL3 basic vector. This plasmid is designated as STMN1-1. Deletion constructs, STMN1-2 (−1343/+325), STMN1-3 (−1264/+325) and STMN1-4 (−1152/+325) were produced by fill-in reaction with DNA polymerase (Klenow fragment) and blunt end ligation.

### Transient transfection and luciferase assays

Transient transfections with various promoter constructs were performed using Lipofectamine (Invitrogen, Carlsbad, CA, USA) according to the manufacturer's instructions. Cells in 6-well dishes were transfected with either 0.1 µg of empty pGL3vector or with promoter-reporter constructs (STMN1-1, STMN1-2, STMN1-3 and STMN1-4), along with 1 ng Renilla luciferase plasmid pRL. In co-transfection experiments, different amounts of Ascl1, Id3 and Hes1 expression plasmids were added. The total amount of DNA added in each transfection was kept constant by addition of an empty control vector. 48 h after transfection, cells were washed with pre-chilled PBS and lysed in passive lysis buffer (Dual Luciferase kit, Promega). Firefly luciferase and Renilla luciferase activities in the cell lysates were measured according to the manufacturer's instructions using a TD 20/20-luminometer (Turner Biosystems, Sunnyvale, CA, USA). Firefly luciferase activity was normalized to the Renilla luciferase activity and reported as relative luciferase activity (RLA).

### Preparation of RNA and Real-Time RT-PCR

The hippocampus was rapidly dissected from each brain, and the hippocampus was divided into Ammon's horn and the dentate gyrus [21]. Preparations of total RNA from the tissues were performed as previously described [22]. Total RNA was isolated from PC12 cells using the RNA Easy Kit (Qiagen, Tokyo, Japan). Each RNA was transcribed to cDNA using reverse transcription reagents (Superscript III; Invitrogen or High-Capacity cDNA Reverse Transcription Kit; Applied Biosystems) according to the manufacturer's instructions.

Real-time RT-PCR was performed on a thermocycler (7900HT Sequence Detection Systems; Applied Biosystems, Foster, CA, USA) with nuclear stain reagents (SYBR Green; Applied Biosystems), according to the manufacturer's instructions. Amplification of PCR products was measured by fluorescence associated with the binding of double-stranded DNA to the SYBR green dye in the reaction mixture. Quantification of each PCR product was expressed relative to GAPDH. The following primers were used: mouse stathmin1; forward, 5′-ccaggcttttgagctgattc; reverse, 5′-gcgtctttcttctgcagctt, mouse GAPDH; forward, 5′-attgtggaagggctcatgacc; reverse, 5′-atgcagggatgatgttctggg, rat stathmin1; forward, 5′-aatggcagaggagaaactgacc; reverse, 5′-cgtgcttgtccttctctcgc, rat Id3; forward, 5′-acatgaaccactgctactcgcg; reverse, 5′-cagaaccacttgaaggtcgagg, rat Hes1; forward, 5′-aaatgacagtgaagcacctccg; reverse, 5′-ttaacgccctcacacgtgg, rat Ascl1; forward, 5′- ttaacctgggctttgccac; reverse, 5′- agcgcgcggatgtattc, rat GAPDH; forward, 5′- gccttctcttgtgacaaagtgg; reverse, 5′- attctcagccttgactgtgcc.

### Chromatin immunoprecipitation (ChIP Assay)

ChIP assays were performed using a ChIP kit (Millipore TM) following the manufacturer's protocol. Briefly, PC12 cells were cross-linked, chromatin was prepared and immunoprecipitated with anti-Ascl1 antibody (Santa Cruz) or with control IgG. Then, immunoprecipitated DNA was eluted and PCR amplified using appropriate primers. For PCR amplification of the E10–11 box (40 cycles), the E10 box (50 cycles) and the negative control (N.C.) (55 cycles), the following primers were used: E10–E11-forward, 5′-tgcctctataagcatattttacgc; E10–E11-reverse, 5′-atttggtctcccaaaagctaaacc; E10-forward, 5′-cagttttcattgtcttgtatgcctg; E10-reverse, 5′-atttggtctcccaaaagctaaacc; N.C.-forward, 5′-gctccgatctcattgttgg; N.C.-reverse, 5′-tcatctagaaacaccgaagcc.

### Immunoblot analysis

Lysates of PC12 cells and of hippocampal dentate gyrus were used directly for western blot analysis as described previously [14], [21]. The following antibodies were employed: rabbit polyclonal anti-stathmin1 (GeneTex). Goat anti-rabbit or anti-mouse IgG conjugated with horseradish peroxidase (Cell Signaling Technology, Beverly, MA, USA) were used as the secondary antibodies. Reaction products were visualized by detection of chemiluminescence using an ECL kit (Amersham Biosciences, Piscataway, NJ, USA). Quantitation of relative band densities was performed by scanning densitometry. All experiments were repeated independently at least three times. PC12 cells were cultured for 1 day at a low cell density, starved of serum for 4 h, and then treated with 100 nM PACAP (PACAP-38) (Peptide Institute, Mino, Osaka, Japan). Cells were harvested at the indicated times after PACAP stimulation.

### Immunohistochemistry

Sections (20 µm) were prepared from frozen brains using a cryostat and thaw-mounted on APS coated slides (Matsunami, Japan) and stored in a tightly closed case at −80°C. The following antibodies were employed: rabbit polyclonal anti-stathmin1 (GeneTex), mouse monoclonal anti-MAP2 (Sigma), anti-Tau (Sigma), and anti-Asc11 (Santa Cruz Biotechnology). Floating sections were incubated with these antibodies overnight at 4°C. Confocal microscopy was performed using an LSM-510 laser scanning microscope (Carl Zeiss, Oberkochen, Germany).

### Quantification of stathmin1 positive cells in the subgranular zone (SGZ) and of dot fibers in the hilus

Stathmin1 cells were counted in coronal sections. The entire DG region of each hippocampus was imaged as a z-series of 20 µm-thick sections. All data analysis was blind to genotype. Statistical analysis was performed using Student's t-test.

After immunolabeling for stathmin1 in equivalent coronal sections, a 20 µm-thick z-series of confocal images was collected in dentate gyrus of hippocampus.

### Double labeling with *in situ* hybridization and immunohistochemistry

The protocol for the *in situ* hybridization (ISH) histochemistry was modified from a previously published method [23]. cDNA fragments of rat PAC_1_ were amplified by RT-PCR using the oligonucleotide primers 5′-cttgtacagaagctgcagtc-3′ (sense) and 5′-ggtgcttgaagtccatagtg-3′ (antisense) and then used as templates for probe synthesis. For double ISH and immunohistochemistry, sections were immunostained followed by ISH. The protocol for immunohistochemistry was based on the published ABC method (Elite ABC kit; Vector, CA, USA) using the rabbit anti-stathmin1 primary antibody at 1∶500. The specificity of the immunohistochemistry was checked by omitting the primary antibody.

### Immunoelectron microscopy

Eight week-old mice were deeply anesthetized with sodium pentobarbital and perfused transcardially with 0.85% physiological saline followed by 0.05% glutaraldehyde and 4% paraformaldehyde in a 0.1 M phosphate buffer (pH 7.4). Brains were removed and post-fixed in the same fixative for 4 h at 4°C, followed by immersion in 30% sucrose in 0.1 M PB overnight at 4°C. 20 µm brains sections were then cut on a cryostat. Immunohistochemistry was performed using free-floating sections according to the ABC method. The anti-stathmin antibodies were used at a dilution of 1∶1000. Biotinylated anti-rabbit IgG (Vectastain Elite) was used as a secondary antibody. Immunoreactivity was visualized with 0.05% diaminobenzidine and 0.01% hydrogen peroxide in 50 mM Tris, pH 7.6. These sections were washed several times in a 0.1 M phosphate buffer (pH 7.4) and after post-fixation with 1% OSO_4_ for 1 h and dehydration they were flat-embedded in Epon 812. Ultrathin sections were viewed without uranyl acetate or lead citrate staining using an H-7000 electron microscope (Hitachi).

### Quantitative RT-PCR assays using Postmortem Brain samples

RNA samples from the dorsolateral prefrontal cortex (DLPFC; Brodmann's area 46) were obtained from the Stanley Medical Research Institute (http://www.stanleyresearch.org/programs/brain_collection.asp). Samples were taken from 35 schizophrenics (26 males, 9 females; mean±SD age, 42.6±8.5 years; postmortem interval (PMI), 31.4±15.5 h; brain pH, 6.5±0.2), 35 bipolar disorder patients (17 males, 18 females; mean±SD age, 45.3±10.5 years; PMI, 37.9±18.3 h; brain pH, 6.4±0.3), and 35 controls (26 males, 9 females; mean±SD age, 44.2±7.6 years; PMI, 29.4±12.9 h; brain pH, 6.6±0.3). Diagnoses were made by applying DSM-IV (the Diagnostic and Statistical Manual of Mental Disorders, Fourth Edition) criteria. All schizophrenic patients were medicated with anti-psychotics. Quantitative RT-PCR analysis was conducted using an ABI7900HT Fast Real-Time PCR System (Applied Biosystems) with TaqMan Gene Expression Assays (Applied Biosystems). All quantitative RT-PCR reactions were performed in triplicate, based on a standard curve method. Detection values are normalized according to the internal controls (GAPDH, ACTB and PGK1). TaqMan probes for STMN1, GAPDH, ACTB, and PGK1 were selected from predesigned TaqMan Gene Expression Assays (AssayID: STMN1, Hs01027516_g1; GAPDH, Hs99999905_m1; ACTB, Hs99999903_m1; PGK, Hs99999906_m1). The Mann-Whitney U test (two–tailed) was used to detect significant changes in target gene expression levels.

### Association Study Subjects

The case-control samples consisted of 1060 unrelated schizophrenic patients (503 men, 557 women; mean age 48.0±13.8 years) and 1060 age- and sex-matched controls (503 men, 557 women; mean age 47.7±13.6 years). All patients had a consensual diagnosis of schizophrenia according to DSM-IV criteria from at least two experienced psychiatrists. Control subjects were recruited from hospital staff and volunteers who showed no present or past evidence of psychoses, during brief interviews by psychiatrists. All participants were recruited from a geographic area located in central Japan. The current study was approved by the Ethics Committees of RIKEN. All participants provided written informed consent.

### SNPs and Genotyping

Three SNPs, rs159522, rs12037513 and rs807061 located in close vicinity of the STMN1 gene were genotyped in this study. SNP genotyping was performed using the TaqMan system (Applied Biosystems, Foster City, CA, USA) according to the recommendations of the manufacturer. PCR was performed using an ABI 9700 thermocycler. Fluorescent signals were analyzed using an ABI7900HT Fast Real-Time PCR System and SDS v2.3 software (Applied Biosystems).

### Statistical Analyses

Concerning the association study, analysis of the significance of differences in mRNA expression between the control group and the chronic stress group was performed using Student's t-test. The allelic and genotypic distributions in the Japanese case-control samples were tested for association by Fisher's exact test. Haplotypic association analysis of Japanese samples was performed using the COCAPHASE program in the UNPHASED v3.0.11 program (http://www.mrc-bsu.cam.ac.uk/personal/frank/software/unphased/) [Dudbridge, 2008]. To estimate the degree of linkage disequilibrium (LD), the standardized disequilibrium coefficient (D′) and the squared correlation coefficient (r2) were calculated using Haploview 4.0 (http://www.broad.mit.edu/mpg/haploview/). The deviation of genotype distributions from the Hardy–Weinberg equilibrium (HWE) was evaluated by the chi-squared test (d.f. = 1). Other results were expressed as the mean±SE, with statistical analysis being performed by a one way ANOVA.

## Results

### Down-regulation of PACAP expression induces up-regulation of stathmin1 expression in the dentate gyrus both *in vivo* and *in vitro*

To detect the genes regulated by PACAP, we searched for gene transcripts that were clearly up-regulated or down-regulated in the dentate gyrus of *Adcyap^−/−^* mice.

**Figure 1:**
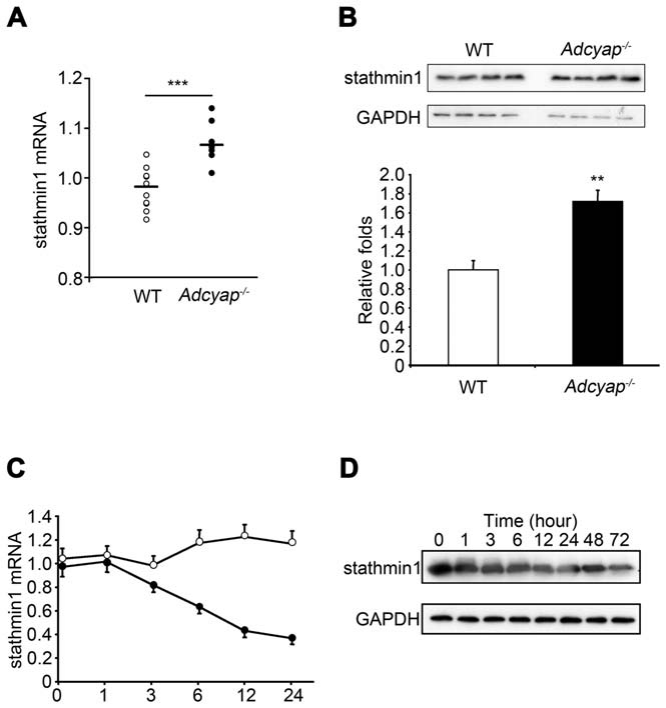
Stathmin1 expression is negatively controlled by PACAP. (A, B) Comparison of stathmin1 expression of wild-type mice to that of *Adcyap^−/−^* mice. (A) Expression level of stathmin1 mRNA in DG was measured by real-time RT-PCR and normalized to the expression of GAPDH. Data represents means±SEM of independent experiments (wild type n = 10, *Adcyap^−/−^* n = 10, ***P = 0.0000496 compared with wild type). (B) Expression of stathmin1 protein in the DG of wild type and *Adcyap^−/−^* mice measured by western blot analysis (upper panel). GAPDH was used as internal control (middle panel). Lower panel shows the ratio of stathmin1 protein level in DG of *Adcyap^−/−^* mice (closed column) to that of wild-type mice (open column). Error bars represent ±SEM. (wild-type n = 4, *Adcyap^−/−^* n = 4, **P = 0.00277 compared with wild-type). (C, D) Kinetic studies of the effect of PACAP signaling on stahmin1 expression levels in PC12 cells. (C) Alteration of stathmin1 mRNA levels by the indicated period of PACAP (100 nM) stimulation was quantified by real-time PCR. Data are expressed as mean percentages ±SEM relative to control values at 0 h. Open circle indicates vehicle treatment. Closed circle represents PACAP treatment. (D) Alteration of stathmin1 protein levels under the indicated period of PACAP (100 nM) stimulation was measured by western blot analysis using an anti-stathmin1 antibody.

The differential display (DD) method showed that 55 cDNA fragments were up-regulated or down-regulated in the dentate gyrus of *Adcyap^−/−^* mice compared with wild-type mice. One of these genes, stathmin1, was subjected to further analysis. Real-time PCR showed that stathmin1 mRNA was markedly increased in the dentate gyrus of *Adcyap^−/−^* mice (Fig. 1A). Increased stathmin1 protein levels in the dentate gyrus of *Adcyap^−/−^* mice were also confirmed by western blot analysis (Fig. 1B). Thus, PACAP deficiency induced elevation of stathmin1 in the dentate gyrus.

**Figure 2:**
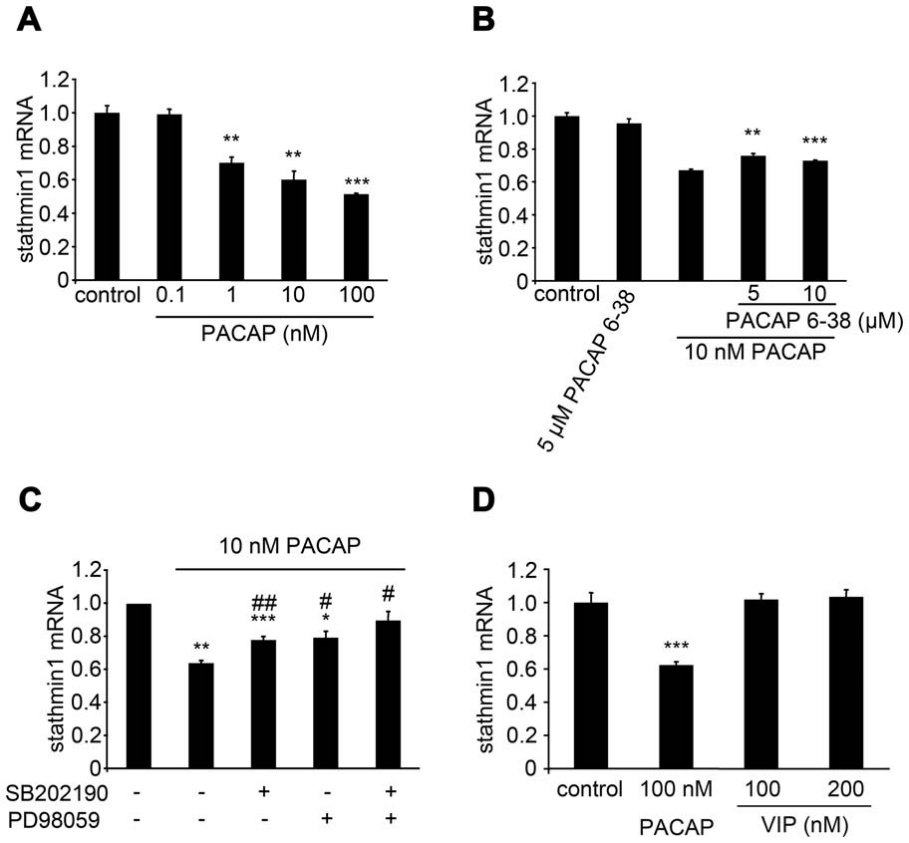
PACAP regulated stathmin1 expression via PAC_1_ in PC12 cells. (A) Alteration of stathmin1 mRNA levels 24 hours after PACAP treatment, at indicated concentrations, was quantified by real-time PCR. Data are expressed as mean percentages ±SEM relative to control values (n = 3, PACAP 1 nM **P = 0.0044, 10 nM **P = 0.0033, 100 nM ***P = 0.0003 compared with control). (B, C) Effect of PACAP signaling pathway inhibitors on the PACAP-induced down-regulation of stathmin1 expression. (B) PC12 cells were treated with 10 nM PACAP for 6 h and incubated with or without the indicated concentration of PAC_1_/VPAC_2_ receptor antagonist, PACAP 6-38 (n = 3, PACAP6-38 5 µM **P = 0.0027, 10 µM ***P = 0.00045 compared with PACAP stimulation alone) and (C) pretreatment of either ERK or p38 inhibitor (n = 3, PACAP stimulation alone **P = 0.0012, SB202190 ***P = 0.0008, ##P = 0.005, PD98059 *P = 0.018, #P = 0.0018, SB202190 & PD98059 #P = 0.013, *compared with each control, #compared with PACAP stimulation alone). Then stathmin1 expression was quantified by real-time PCR. Data are expressed as mean ratios ±SEM relative to control values. (D) Alteration of stathmin1 mRNA levels in PC12 cells, 6 hours after PACAP or VIP treatment at the indicated concentrations, was quantified by real-time PCR. Data are expressed as mean percentages ±SEM relative to control values (n = 3, PACAP 100 nM ***P = 7.08E^−07^ compared with control).

We then examined whether the *in vivo* changes described above could be reproduced *in vitro* using PC12 cells. Stathmin1 mRNA levels were decreased 3 hours after PACAP stimulation, and expression continued to decrease over the next 24 hours (Fig. 1C). PACAP stimulation of PC12 cells caused stathmin1 protein levels to decrease (Fig. 1D), and also caused a dose-dependent decrease of stathmin1 mRNA levels (Fig. 2A). The decrease of stathmin1 expression by PACAP stimulation was slightly, but statistical significantly, inhibited by pretreatment with a PAC_1_/VPAC_2_ receptor antagonist (PACAP6-38) (Fig. 2B). We did not perform the Western blot analysis, because we assumed that this small difference would not be detectable due to the limitation of its sensitivity. Pretreatment with a p38 antagonist (SB202190) or an ERK antagonist (PD98059) also inhibited the decrease of stathmin1 expression by PACAP (Fig. 2C). Co-administration of SB202190 and PD98059 strongly inhibited the effect of PACAP (Fig. 2C). The p38 and ERK are key elements of the PACAP signaling pathway (supplementary Fig. S1). On the other hand, VIP did not decrease stathmin1 expression (Fig. 2D). These results indicate that PACAP inhibits stathmin1 expression in PC12 cells. Furthermore, we showed that PACAP regulates stathmin1 expression via the PAC_1_ receptor in neurons of the dentate gyrus subgranular zone, as described below.

### Up-regulation of stathmin1 induces abnormal axonal arborization in neurons of the dentate gyrus subgranular zone

#### Stathmin1 is mainly localized in subgranular zone neurons with prominent localization in cell processes

**Figure 3:**
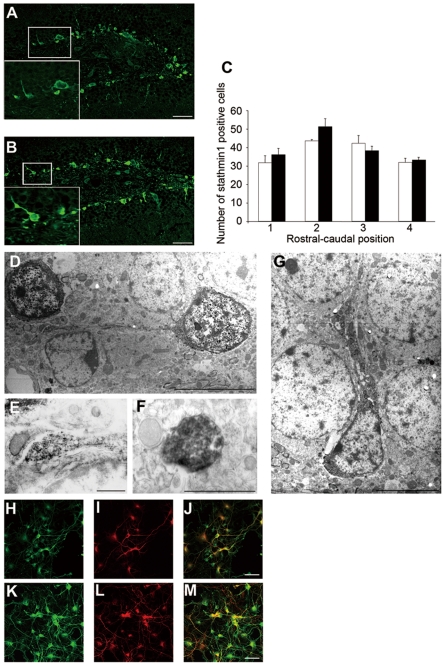
Stathmin1 expression in the axon and dendrite. (A–B) Immunohistochemical analysis of stathmin1 in the DG. Immunohistochemistry of stathmin1 using wild-type mice (A) and *Adcyap^−/−^* mice (B). The small photograph is at high magnification. Bars = 50 µm. (C) The number of stathmin1 positive cells in the SGZ in wild-type (open column) and *Adcyap^−/−^* mice (closed column) were counted in a set of four coronal sections along the rostral-caudal range of each hippocampus/DG (n = 3). (D–G) Immunoelectron microscopic analysis of stathmin1-expressing processes in the SGZ of *Adcyap^−/−^* mice. (D, E) Stathmin1 positive neurites originating from SGZ neurons extended to the polymorphic layer (D, bar = 5 µm) and the neurites had the morphology of axon fibers (E, bar = 1 µm). (F) Stathmin1 positive dot-like fibers in the polymorphic layer identified as the axon fibers. Bar = 1 µm (G) Thick processes found in the granular cell layer could often be traced to the stathmin1 positive cell soma. Bar = 5 µm (H, K) Localization of stathmin1 in the rat hippocampal primary neurons. The same cells as in (H) and (K) stained with MAP2 antibody (I) or anti-Tau antibody (L). (J) and (M) represent the merged image of (H) with (I), and (K) with (L), respectively. Bars = 50 µm.

**Figure 4:**
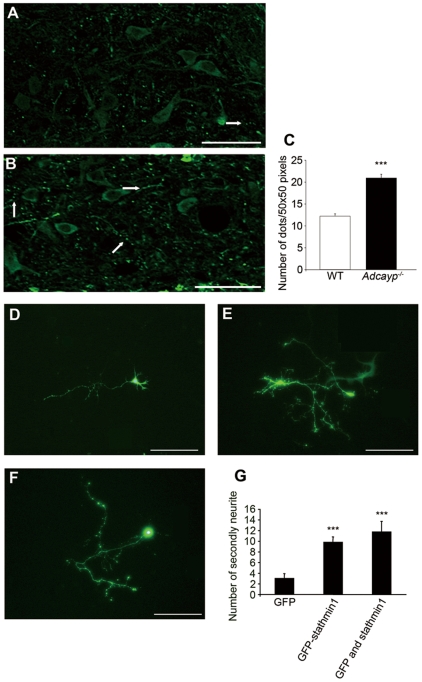
Stathmin1 over-expression in neurons causes abnormally pronounced arborization of axon fibers. (A, B) Immunohistochemical analysis of stathmin1 in the polymorphic layer. Immunohistochemistry of stathmin1 using wild-type mice (A) and *Adcyap^−/−^* mice (B). White arrows indicate varicosities. Bars = 50 µm. (C) The number of stathmin1 positive dot-like fibers in the polymorphic layer were counted (n = 3, ***P = 3.4E^−12^ compared with wild-type). Error bars represent ±SEM. (D–F) Morphology of hippocampal primary neurons transfected with stathmin1. Over-expression of GFP (D), GFP-stathmin1 (E) or stathmin1 and GFP (F). The neurons over-expressing stathmin1 have abnormally pronounced sprouting of axon fibers. Bars = 50 µm. (G) The number of secondary neurites from axons was increased by over-expression of stathmin1 (GFP; n = 10, GFP-stathmin1; n = 10, ***P = 2.5E^−5^, GFP and stathmin1; n = 6, ***P = 0.00022 compared with GFP). Error bars represent ±SEM.

Immunohistochemistry for stathmin1 in the dentate gyrus of wild-type mice showed that cells expressing stathmin1 were preferentially localized in the innermost part of the granular cell layer, the so-called subgranular zone (SGZ) where neurogenesis of granular cells occurs in adults (Fig. 3A, B). A large number of cells in the SGZ expressed stathmin1. Immunohistochemical analysis also revealed that there were two types of stathmin1 containing processes; thick processes and dot-like processes. Thick processes in the granular cell layer could often be traced to the soma of stathmin1-positive cells (Fig. 3A, B). Numerous dot-like processes were exclusively found in the polymorphic layer and often formed varicosities (Fig. 4A, B white arrows). Immunoelectron microscopy established that stathmin1 positive cells extend neurites to the hilus, and that these neurites were axons, judging by their morphology (Fig. 3D, E). Thus, the dot-like processes were the fragments of axons (Fig. 3F), while the thick processes to the granular cell layer were dendrites (Fig. 3G). Similarly, primary cultured neurons expressed stathmin1 in the soma and processes under normal conditions. MAP2 and Tau staining established that stathmin1 was expressed in dendrites (Fig. 3H, I, J) and in axons (Fig. 3K, L, M).

#### Elevation of stathmin1 in dentate gyrus neurons causes abnormal axonal arborization

Immunoreactivity for stathmin1 was significantly increased in the SGZ neurons of *Adcyap^−/−^* mice (Fig. 3A, B), although the actual number of immunoreactive cells was similar in mutant and wild-type mice (Fig. 3C). The number of dot-like immunoreactive fibers was significantly increased in the polymorphic layer of *Adcyap^−/−^* mice compared with wild-type mice (Fig. 4A–C). These findings show that increased expression of stathmin1 in the SGZ neurons led to pronounced arborization of the axons of SGZ neurons. Therefore, we attempted to clarify whether this *in vivo* event could be duplicated *in vitro* by using hippocampal primary cultured neurons. Over-expression of stathmin1 caused dramatic changes of axon fibers. As shown in Figure 4E and 4F, arborization of axon fibers was markedly increased by stathmin1 over-expression compared with that in normal primary cultured neurons (Fig. 4D). The number of secondly neurites from axons was also increased following over-expression of stathmin1 (Fig. 4G). Thus, it was concluded that an increase of stathmin1 expression in SGZ neurons leads to abnormal axonal arborization.

### Molecular mechanism of stathmin1 regulation by PACAP

#### PACAP regulates stathmin1 expression via the PAC_1_ receptor in SGZ neurons

**Figure 5:**
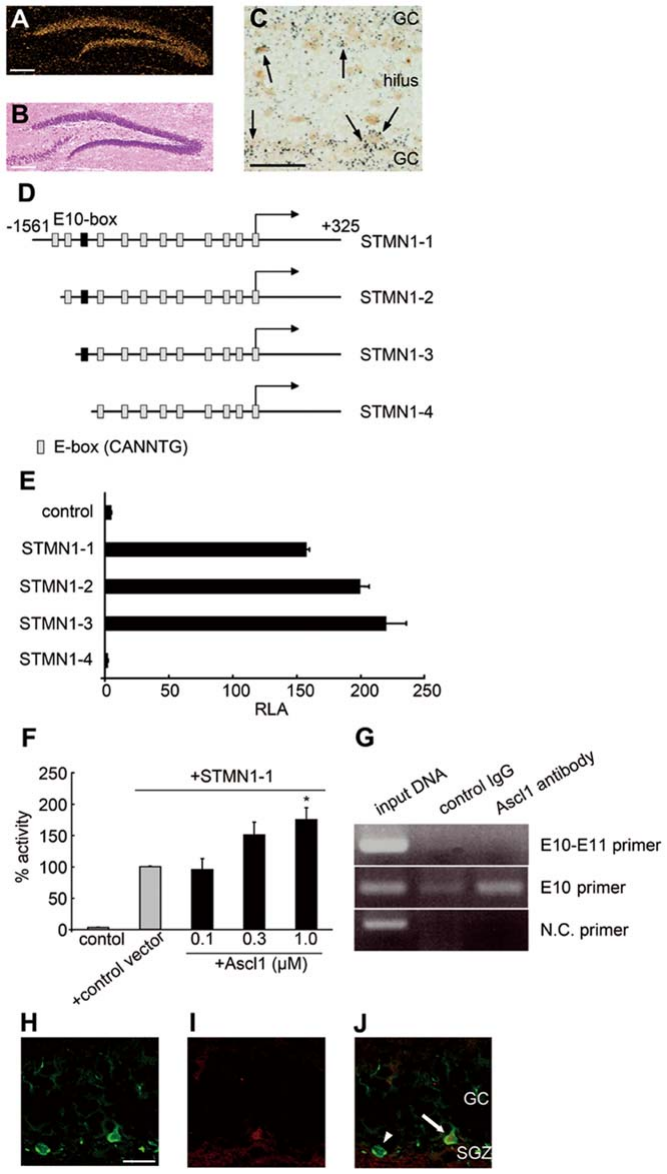
PACAP inhibits stathmin1 by up-regulation of Ascl1 using p38 and ERK signals. (A) Localization of stathmin1 and PAC_1_ expressing neurons in the DG of wild-type mice. (B) Hippocampal section stained with hematoxylin and eosin. Bars = 100 µm. (C) shows the localization of stathmin1 and PAC_1_ expressing neurons in the same section (arrows). Stathmin1 is visualized by immunohistochemistry (brown) and PAC_1_ mRNA by *in situ* hybridization (black grains). Bars = 50 µm. (D–J) Involvement of Ascl1 in the promoter activity of stathmin1. (D) Schematic diagram of the 1.8 kb rat stathmin1 5′ genomic DNA fragments for the luciferase activity assay. These vectors posses the 5′ regulatory region of stathmin1, the first exon and partial sequence of the first intron. The different promoter constructs used in this study are shown. The relative location (not drawn to scale) of putative E-boxes, and the transcription start site (arrow at +1) are shown. (E) PC12 cells were transfected in 6-well plates with the indicated stathmin1 promoter-luciferase plasmids and the Renilla luciferase plasmid. Firefly and Renilla luciferase activities were measured using a Dual Luciferase Reporter Assay System (n = 3). Luciferase activity was measured and data (means±SEM) are shown as relative luciferase activity (RLA). (F) Activation of the STMN1-1 promoter by Ascl1. STMN1-1 plasmid and 1 µg of expression vector, which are mixed with empty vector pCI and the indicated amounts of pCI-Ascl1, were transfected in triplicate, with 0.2 µg. 48 h after PC12 cell transfection, the cells were collected and the RLA was determined. Data (mean±SEM) show representative results of least three experiments, *P = 0.0165. (G) Sheared chromatin from PC12 cells was immunoprecipitated with the appropriate antibody (anti-Ascl1, control rat anti-IgG). Then DNA sequences bound to precipitated proteins were isolated. Immunoprecipitated DNA was used as template in PCR using E10 primers, E10–E11 primers or negative control primers. (H–J) Localization of stathmin1 (H) and Ascl1 (I) were demonstrated on the same DG section of wild-type mice. Arrows indicate SGZ neurons containing both stathmin1 and Ascl1. Arrow head represents a neuron expressing stathmin1 alone. Bar = 10µm.

If PACAP directly regulates stathmin1 expression *in vivo*, SGZ neurons should express PAC_1_. In fact, strong expression of PAC_1_ mRNA was identified throughout the entire granule cell layer, including the SGZ (Fig. 5A). Figure 5C shows the localization of stathmin1-expressing neurons (brown) and PAC_1_ mRNA-expressing neurons (black grains) in the same section of the dentate gyrus. As indicated by arrows, SGZ neurons were identified that expressed both stathmin1 protein and PAC_1_ mRNA (Fig. 5C). Thus, stathmin1-expressing neurons in the SGZ also expressed PAC_1_.

#### Stathmin1 gene promoter activity is regulated by basic helix loop helix (bHLH) proteins

Using to perform a BLAST search, we identified the genomic sequence of rat stathmin1 in a chromosome 5 contig. We then performed PCR amplification of a 1885 bp genomic DNA fragment that consisted of 1561 nt upstream of the stathmin1 transcription start site (+1), exon 1, and part of intron 1(+325). This fragment was sequenced and subcloned into the pGL3 luciferase reporter vector. We also analyzed this fragment for transcription factor-binding sites using the DNAsis program. The 1.8 kbp rat stathmin1 5′ genomic sequence contained 12 (E1–E12) putative E boxes (CANNTG), which are potential binding sites for bHLH proteins, including neuronal transcriptional activators (Fig. 5D). To investigate the promoter activity of stathmin1, we constructed several expression plasmids for the luciferase reporter assay (Fig. 5D). We examined the activity of each construct using the luciferase assay, after transient transfection in PC12 cells. Constructs with E10 (such as STMN1-1, STMN1-2 and STMN1-3) showed a high level of luciferase activity compared with control cells, but constructs with a stathmin1 promoter lacking the E10 box (such as STMN1-4) did not show luciferase activity (Fig. 5E). Therefore, the E10 box was found to be a key motif that regulates stathmin1 expression through bHLH factors.

#### An activating bHLH protein, Ascl1, activates the stathmin1 promoter

Among activating bHLH proteins, we found that Ascl1 activated the stathmin1 promoter. Namely, co-transfection of PC12 cells with the stathmin1-promoter plasmid and the Ascl1 expression plasmid induced a dose-dependent increase of luciferase activity compared with transfection of the stathmin1 promoter plasmid alone (Fig. 5F). To examine whether endogenous Ascl1 protein in PC12 cells could bind to the stathmin1 promoter sequence, sheared chromatin was immunoprecipitated with anti-Ascl1 antibody or with control IgG, followed by PCR amplification of the corresponding DNA regions using stathmin1 promoter specific primers. Analysis of amplified DNA showed that more sequences were amplified by primers flanking the E10 box compared with primers flanking the E10–E11 boxes (Fig. 5G). In addition, co-localization of Ascl1 and stathmin1 in SGZ neurons was demonstrated by immunohistochemistry (Fig. 5H, I, J). These results established that endogenous Ascl1 protein could bind to the stathmin1 promoter and act as a major regulator of stathmin1 promoter activity.

#### Inhibitory bHLH proteins, Hes1 and Id3, show increased expression after PACAP stimulation

**Figure 6:**
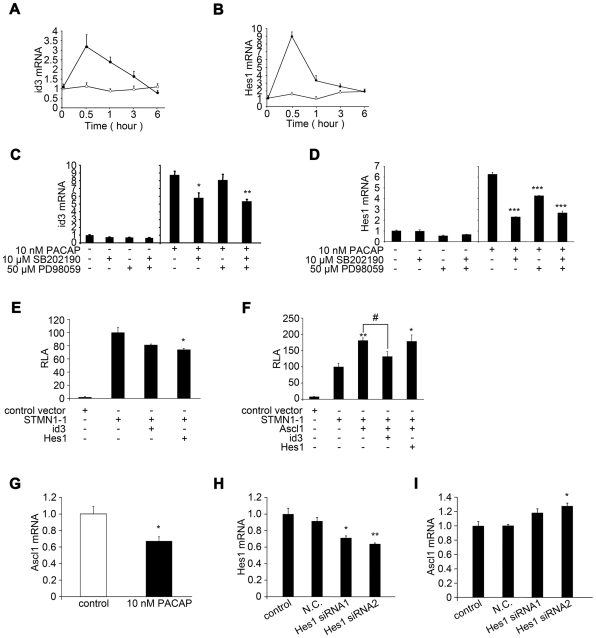
Hes1 and Id3, which are regulated by PACAP, inhibit Ascl1 function. (A, B) Kinetic study of the effect of PACAP on the expression of Id3 (A) and Hes1 (B) mRNA. PC12 cells were treated with 10 nM PACAP for the indicated times and Id3 and Hes1 expression was quantified by real-time PCR. Data are expressed as mean percentages ±SEM relative to control values at 0 h. Open circle indicates vehicle treatment. Closed circle represents PACAP treatment. (C, D) Up-regulation of Id3 expression was inhibited by treatment with a p38 inhibitor (SB202190) (n = 3, SB202190 *P = 0.0202, SB202190 & PD98059 **P = 0.0032 compared with control) (C), and expression of Hes1 mRNA was inhibited by treatment with p38 (SB202190) and ERK (PD98059) inhibitors (n = 3, SB202190 ***P = 1.68×10^−5^, PD98059 *** P = 0.000252, SB202190 & PD98059 *** P = 8.98×10^−5^ compared with control) (D). PC12 cells were incubated with each inhibitor for 1 h, then treated or not with 10 nM PACAP for 0.5 h. Id3 and Hes1 expression were quantified by real-time PCR. Data are expressed as mean ratios ±SEM relative to control values for inhibitor alone. (E) Effect of over-expression of Id3 or Hes1 on the promoter activity of stathmin1. PC12 cells were transfected with STMN1-1 promoter-luciferase plasmid alone or with Id3 expression plasmid, pCI-Id3 or Hes1 expression plasmid, pCI-Hes1. Co-transfection of Id3 or Hes1 expression plasmids inhibited the activity of STMN1-1 promoter plasmid (n = 3, +pCI- Id3 P = 0.0743, +pCI-Hes1 *P = 0.0305 compared with +control vector). (F) Id3, but not Hes1, inhibited the activity of STMN1-1 promoter increased by co-transfection of Ascl1 expression plasmid, pCI-Ascl1 (n = 3, +pCI-neo +Ascl1 **P = 0.00385, +pCI-Hes1 +Ascl1 *P = 0.025 compared with +pCI-neo, +pCI- Id3 +Ascl1 # P = 0.0454 compared with +pCI-neo +Ascl1). (G) The effect of PACAP on the expression of Ascl1 mRNA in PC12 cells. Ascl1 expression was quantified by real-time PCR (n = 3, *P = 0.048 compared with control). Data are expressed as mean percentages ±SEM relative to control. (H, I) Effects of down-regulation of Hes1 on the expression level of Ascl1 mRNA. The effect of siRNAs for Hes1 are shown in (H) (n = 3, siRNA1 *P = 0.0159, siRNA2 **P = 0.00636 compared with control). The expression level of Ascl1 was quantified by real-time PCR (n = 3, siRNA1 P = 0.076, siRNA2 *P = 0.0162 compared with control). Data are expressed as mean ratio ±SEM relative to control values.

As mentioned above, both PACAP and Ascl1 regulate stathmin1 expression. In addition, inhibitory bHLH proteins (Hes family proteins and Id family proteins) are known to inhibit the bHLH proteins. We detected an increase of Hes1 and Id3 expression after PACAP stimulation (Fig. 6A, B). To establish the pathway involved, we next examined the effect of PACAP on Hes1 and Id3 expression *in vitro*. Hes1 and Id3 expression increased rapidly after PACAP stimulation, indicating that expression of bHLH inhibitors (Hes1 and Id3) was induced by PACAP stimulation. Inhibition of elements of the PACAP signaling pathway, such as p38 and ERK, suppresses the effect of PACAP on stathmin1 expression. Therefore, we next examined whether the ERK or p38 pathway regulates the induction of Hes1 and Id3 after PACAP stimulation. As a result, the increase of Id3 mRNA levels in response to PACAP stimulation was inhibited by a p38 inhibitor (SB202190), but not by an ERK inhibitor (PD98059) (Fig. 6C). Induction of Hes1 mRNA by PACAP stimulation was inhibited by an ERK inhibitor and by a p38 inhibitor. Furthermore, co-administration of p38 and ERK inhibitors strongly inhibited the induction of Hes1 mRNA by PACAP stimulation (Fig. 6D). Administration of SB202190 or PD98059 alone did not affect the expression of Hes1 or Id3 largely. These results were represented as a relative value to inhibitor alone. These findings suggested that Hes1 expression is regulated by both the PACAP-ERK and PACAP-p38 pathways, whereas Id3 expression is mainly controlled by the PACAP-p38 pathway.

#### Hes1 and Id3 suppress stathmin1 promoter activity via Ascl1 inhibition

To elucidate whether the stathmin1 promoter was regulated by Hes1 or Id3, PC12 cells were co-transfected with a stathmin1 promoter plasmid and a Hes1 expression plasmid or an Id3 expression plasmid with or without an Ascl1 expression plasmid. Even without exogenous Ascl1 expression, a high level of luciferase activity, due to the stathmin1 promoter, was detected (Fig. 6E). Expression of Hes1 or Id3 in these cells inhibited luciferase activity related to the stathmin1 promoter (Fig. 6E), showing that Hes1 and Id3 blocked stathmin1 promoter activity through endogenous Ascl1. In PC12 cells transfected with the stathmin1 promoter plasmid and the Ascl1 expression plasmid, luciferase activity was higher than that in PC12 cells without the Ascl1 expression plasmid (Fig. 6F). Id3 expression in these cells inhibited the up-regulation of stathmin1 promoter luciferase activity, while Hes1 expression failed to reduce the luciferase activity induced by exogenous Ascl1 (Fig. 6F). These findings suggested that Id3 inhibits activation of the stathmin1 promoter by both exogenous and endogenous Ascl1, while Hes1 only blocked the effect of endogenous Ascl1. Thus, it is likely that Id3 regulates Ascl1 at the protein level, while Hes1 regulates Ascl1 transcription. If so, inhibition of Hes1 expression should increase the transcription of Ascl1. As expected, up-regulation of Hes1 in PC12 cells by PACAP stimulation led to inhibition of Ascl1 expression (Fig. 6G). And a reduction of Hes1 expression also resulted in an elevation of Ascl1 expression to 1.2-fold the control level (Fig. 6H, I). These results indicate that Ascl1, which controls stathmin1 expression, was functionally regulated by Id3 and quantitatively regulated by Hes1, in response to PACAP signaling.

### Stathmin1 expression is increased in the brains of patients with schizophrenia

**Figure 7:**
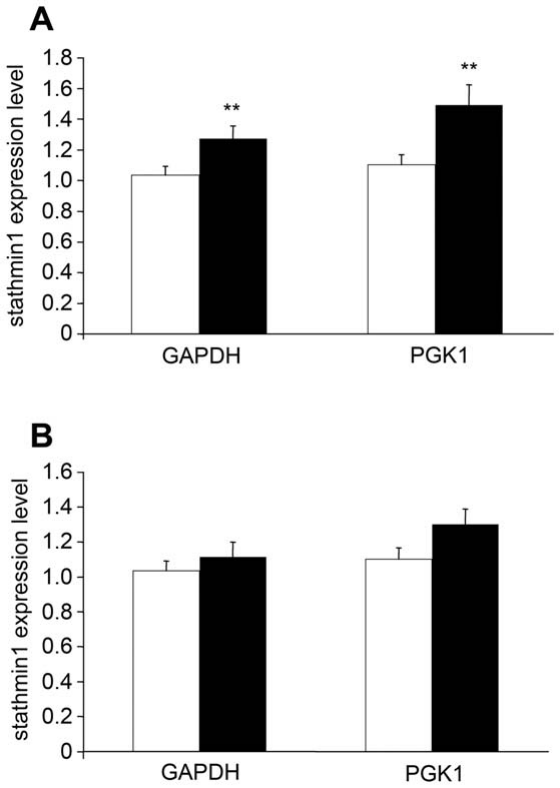
Expression of stathmin1 is increased in the brain of schizophrenia patients. Comparison of mRNA levels of stathmin1 in the postmortem brain of control subjects (open column) and schizophrenia subjects (closed column) (A), or bipolar disorder subjects (B). The relative expression levels of the gene, normalized to GAPDH or PGK1, are presented. The expression of stathmin1 normalized to GAPDH (Mann-Whitney test, p = 0.0082) and PGK1 (Mann-Whitney test, p = 0.0047) were significantly increased in the schizophrenia group, compared with the control group.

*Adcyap^−/−^* mice are known to show behavioral abnormalities, some of which might have potential relevance to mental disorders such as schizophrenia [12]. The present study demonstrated that PACAP inhibits stathmin1 expression both *in vivo* and *in vitro*. Therefore, we examined whether stathmin1 mRNA levels were altered in the brains of patients with schizophrenia. RT-PCR with stathmin1 primers (after normalization with GAPDH and PGK1) showed that the stathmin1 mRNA level was significantly increased in schizophrenic patients compared with age-matched controls (Fig. 7A). In contrast, stathmin1 was not significantly increased in the brains of patients with bipolar disorder (Fig. 7B).

## Discussion

We searched for genes that showed marked changes of expression in the dentate gyrus of *Adcyap^−/−^* mice, because PACAP may play a role in schizophrenia and the PAC_1_ receptor is exclusively expressed in the dentate gyrus of the hippocampus. As a result, we identified alterations in the expression of stathmin1. This study provides the first *in vivo* and *in vitro* demonstration that: (1) PACAP suppresses stathmin1 expression; (2) increased expression of stathmin1 in SGZ neurons, due to PACAP deficiency, causes abnormal axonal sprouting and stathmin1 over-expression in primary cultured hippocampal neurons induces abnormal arborization; (3) ascl1 regulates stathmin1 expression; (4) up-regulation of Id3 and Hes1 by PACAP stimulation inhibits Ascl1; and (5) stathmin1 expression was increased in the brains of patients with schizophrenia.

### Increased stathmin1 expression in SGZ neurons due to PACAP deficiency induces abnormal neurite outgrowth

There are no significant data about the morphological changes of neurons in the hippocampus in postmortem brains of schizophrenia patients. A tendency for dendrite lengths to be shortened has been reported and the density of spines was reduced in the cortex of schizophrenia patients [24]. What happens in the brain after disturbance of the PACAP-PAC_1_-stathmin1 cascade? To investigate this point, the functions of stathmin1 will need to be explored, because, to date, little is known. It has been reported that stathmin1 is required for the induction of long-term potentiation (LTP) in afferent inputs to the amygdala and is essential for regulating both innate and learned fear [25], [26], [27]. Cardinaux *et al.* reported that brain-derived neurotrophic factor (BDNF) stimulates the phosphorylation of stathmin1 [28]. Rowlands *et al.* showed that stathmin1 is expressed in proliferating cells, but not in resting cells [29]. These findings suggest an important role of stathmin1 in development, but the details of its actions in the brain are still obscure. In the present study, stathmin1 expression was demonstrated in neurons of SGZ where neurogenesis is ongoing, even in adulthood. The SGZ contains neurons in various stages of neurogenesis and a large number of SGZ neurons expressed stathmin1, suggesting that it is expressed at a specific stage of neurogenesis. Nestin is a marker of proliferating neurons [30], but stathmin1 and nestin were localized in different populations of neurons. PSA-NCAM and doublecortin (DCX) are markers of migrating neurons [31], [32], whereas NeuN is a marker of mature neurons [33], and stathmin1 was co-localized with these markers of neuronal differentiation (supplementary Fig. S2). These results indicate that stathmin1 is not involved in the proliferation of SGZ neurons, but influences their differentiation. Moreover, we found stathmin1-expressing neurons in the rostral migratory system, olfactory bulb, and piriform cortex (data not shown) where proliferation and differentiation of neurons also occur during adulthood. Furthermore, PAC_1_ was co-localized with stathmin1 in the olfactory bulb and piriform cortex (data not shown). These findings suggest that PACAP signaling regulates stathmin1 expression and controls neurogenesis.

Furthermore, we explored the role of stathmin1 in neurite outgrowth, because over-expression of stathmin1 by hippocampal neurons caused pronounced aberrant arborization of axons both *in vivo* and *in vitro*. Stathmin1 knock-out mice show normal neuronal morphology [25], while over-expression of stathmin1 after reduction of PACAP signal induces abnormal arborization of axons. These findings suggested that stathmin1-related proteins, such as SCG10 [34], can compensate for the lack of stathmin1 activity in developing cells, but destabilization of microtubules (MTs) by an increase of stathmin1 is unavoidable because it interacts directly with tubulin [35], [36]. It can be suggested that the regulation of MT stability in the early stage of neurogenesis is important for the correct formation of neurons. Although we detected increased *in vivo* arborization of axons in the polymorphic layer, no obvious morphological changes of the dendrites were observed. Further investigation is needed to elucidate why stathmin1 expression causes the sprouting of axon fibers only. Previously, Inokuchi *et al.* reported the curious finding that both over-expression and down-regulation of stathmin1 inhibited the outgrowth of dendrites from cultured Purkinje cells [35], [37]. However, even they found no morphological alterations of the dendrites of Purkinje cells in stathmin1 knock-out mice, and explained this as being due to the compensatory effect of stathmin1-related molecules [38]. Although it is difficult to explain the discrepancy between the present and previous findings, it could be attributable to differences among the neurons studied. Taken together, the data suggest that regulation of proteins related to MT stability regulates axon or dendrite formation and that these proteins are modulated by neurotrophic factors, such as PACAP.

### Molecular mechanism for PACAP inhibition of stathmin1 expression in SGZ neurons

It was previously shown that the expression of stathmin1 in PC-3-M cells is predominantly mediated through the E2F family of transcription factors [39]. From BLAST search, it indicates that the stathmin1 promoter region contains multiple bHLH binding sites. In addition, human and mouse stathmin1 promoters have several E boxes, and these transcriptional sites should be key motifs for regulating stathmin1 expression. As mentioned above, stathmin1 was co-localized with DCX and PSA-NCAM in the SGZ. Neuronal transcription factors from the family, such as Ascl1 and NeuroD, are expressed before the migration stage, which is when expression of DCX or PSA-NCAM occurs [40]. We examined the effect of Ascl1 and NeuroD on the stathmin1 promoter by using a luciferase assay. Ascl1 was shown to enhance luciferase activity related to the stathmin1 promoter, but NeuroD did not (data not shown). Ascl1 and all the members of the bHLH family heterodimerize through their HLH domain with ubiquitously expressed bHLH E proteins, such as E2A gene products (E12 and E47). Heterodimers bind to DNA through their basic domain and activate the transcription of genes that have an E box in the promoter region [41]. Thus, it can be suggested that heterodimers of Ascl1 and E47 bind to the E10 box motif of the stathmin1 promoter region and activate stathmin1 expression.

The present study suggested the molecular cascade by which PACAP inhibits stathmin1 expression (Fig. 2, 5 and 6). Neuronal differentiation is known to be not only regulated by transcriptional bHLH activators, but also by inhibitory bHLH proteins, such as the Hes and Id family of repressors. We found that Hes1 and Id3 were rapidly up-regulated by PACAP, and activation of Ascl1 was inhibited by both Hes1 and Id3. Hes1 expression was increased via both the PACAP-PAC_1_-ERK and PACAP-PAC_1_-p38 pathways, whereas Id3 was up-regulated via the PACAP-PAC_1_-p38 pathway only. We also detected different mechanisms for the inhibition of Ascl1 by Hes1 and Id3. First, Hes1 regulates the transcription of Ascl1. We observed that Hes1 was up-regulated by PACAP, but Hes5 was unchanged (data not shown). The Hes family can inhibit gene expression either by directly binding (as homodimers) to cognate-binding elements known as N-boxes (CACNAG) or indirectly by dominant-negative regulation (i.e., forming non-functional heterodimers with transcriptional activators) [41]. It has been shown that the repressive effect of Hes1 is directly mediated by its binding to a class C site in the hAsh1 promoter [42]. Hes1 is thought to regulate the onset of neuronal differentiation, at least partly, by repressing the transcription of downstream positive factors, such as the neuronal commitment gene, Ascl1. Therefore, Hes1 (which was up-regulated by PACAP) binds the Ascl1 promoter and inhibits Ascl1 expression. On the other hand, Id3 regulates the function of Ascl1 protein. Id proteins have been identified as negative regulators of bHLH transcription factors and Id1 through Id4 have been characterized in mammals. It was previously shown that Id3 is a cAMP-responsive gene, the up-regulation of which could be involved in PACAP signaling [43]. We observed that Id3 was up-regulated by PACAP stimulation, but Id1, Id2 and Id4 increased at a later phase (data not shown). These transcriptional regulators inhibit bHLH factors by sequestering them in inactive heterodimers, which are unable to bind with DNA due to the absence of a basic DNA-binding region in the Id proteins. In addition, previous findings have indicated that Id1 and E protein levels not only regulate the transcription but also the stability of Ascl1 [44]. Thus, our results suggest that Id3 binds E protein and suppresses Ascl1.

The present study indicated that PACAP signaling increased the levels of Hes1 and Id3, following these inhibitory bHLH proteins decreased the expression of stathmin1 (supplementary Fig. S3).

### Increased stathmin1 expression in SGZ neurons due to PACAP deficiency and psychiatric illness

PACAP and PAC_1_ knock-out mice show abnormal behavior resembling psychiatric disorders [5] and some SNPs in the vicinity of the PACAP gene locus are associated with schizophrenia. These findings suggested that the brains of schizophrenic patients may be deficient in PACAP and PAC_1_, resulting in increased expression of stathmin1 in the SGZ neurons of the dentate gyrus. In addition, up-regulation of stathmin1 has previously been implicated in schizophrenia pathogenesis from the results of scanning and from comparing the anterior cingulate cortex gray matter proteomes between schizophrenia and control post-mortem human tissue using two-dimensional gel electrophoresis [45]. As expected, the present study revealed an increase in the expression of stathmin1 mRNA in the brains of schizophrenic patients, but not in the brains of patients with bipolar disorder. The fold-index indicated about 1.5 (schizophrenia/control), which corresponded well to the level (1.8) in the report above. However, no SNPs displayed any allelic, genotypic, or haplotypic associations with schizophrenia (supplementary Table S1).

Recent studies have provided reliable evidence that schizophrenia is a neurodevelopmental disease [46], [47], [48]. The present study revealed that PACAP deficiency causes increased stathmin1 expression in SGZ neurons and leads to abnormal sprouting of their axons, showing that PACAP deficiency and consequently over-expression of stathmin1 in SGZ neurons could lead to a disturbance of the neural circuits in the dentate gyrus. This raises the possibility that abnormal expression of PACAP and stathmin1 could interfere with dentate gyrus functions, leading to an increased susceptibility to psychiatric illnesses.

## Supporting Information

Figure S1 P38 or ERK activation in PC12 cells in response to PACAP stimulation. PC12 cells were treated with 100 nM PACAP. After the stimulation, cells were lysed at each indicated times. Upper 2 panels: Activations of p38 were detected by immunoblotting analysis using the phosphospecific anti-p38 antibody. To control for loading, Western blotting analyses of lysates were performed with anti-p38 antibody. Lower 2 panels: Activations of ERK were also detected by immunoblotting analysis using the phosphospecific anti-ERK antibody. To control for loading, Western blotting analyses of lysates were performed with anti-ERK antibody. Used antibodies as follows; anti-P38 antibody (Cell Signaling. rabbit polyclonal, 1∶1000 dilution), anti-phosopholylated P38 antibody (Cell Signaling. rabbit polyclonal, 1∶1000 dilution), anti-ERK antibody (Cell Signaling. rabbit polyclonal, 1∶1000 dilution), anti-phosopholylated ERK antibody (Cell Signaling. rabbit polyclonal, 1∶1000 dilution). Note: Both P38 and ERK were activated at 5–10 minutes after PACAP stimulation.
(0.40 MB TIF)

Figure S2 Localisation of stathmin1 with markers of various stages in neurogenesis in dentate gyrus of 8 week-mice. A–C. Mouse hippocampus (dentate gyrus) was stained with anti-stathmin1 and anti-nestin antibodies (A, stathmin1: green, B, nestin: red, C, merged image). D–G. With anti-stathmin1 and anti-PSA-NCAM antibodies (D,E, stathmin1: green, D,F, PSA-NCAM: red, D,G, merged image). E–G: Higher magnification of D. scale bar = 50mm. H–K. With anti-stathmin1 and anti-doublecortin (DCX) antibodies (H,I, stathmin1: green, H,J, DCX: red, H,K, merged image). I–K: Higher magnification of H. scale bar = 50mm. L. Merged image with anti-stathmin1 and anti-NeuN antibodies (stathmin1: green, NeuN: red) scale bar = 50mm. Mice under deep pentobarbital anesthesia were perfused transcardially with 30–50 ml of 4% paraformaldehyde solution. Brain was removed and infused with 30% sucrose overnight at 4oC. After blocking with 5% bovine serum albumin, each sections were incubated overnight at 4 oC with an anti-stathmin1 antibody (GeneTex, Inc. rabbit-polyclonal, 1∶1000 dilution) and either anti-nestin (Becton Dickinson. Mouse-monoclonal, 1∶1000 dilution), anti-PSA-NCAM (abcys. Mouse-polyclonal, 1∶1000 dilution), anti-doublecortin (DCX) (Santa Cruz Bio. Goat-polyclonal, 1∶1000) or anti-NeuN (Chemicon. mouse-monoclonal, 1∶1000 dilution) antibody in 0.01 M PBS containing 0.3% Triton X-100 and 5% BSA. Next, the sections were treated with fluorescent dye (Alexa Fluor 488)-conjugated donkey anti-rabbit IgG (1∶1000 dilution), fluorescent dye (Alexa Fluor 568)-conjugated goat anti-mouse IgG (1∶1000 dilution), and donkey anti-goat IgG (1∶1000) as the secondary antibodies for 1 h at RT in 0.01 M PBS containing 5% BSA. Each sections were observed under a confocal microscope (LSM510, Carl Zeiss).
(1.76 MB TIF)

Figure S3 Schematic drawing of the molecular pathway of PACAP regulation on stathmin1 expression shown in this study. A schematic representation of the pathway which PACAP regulates expression of stathmin1 by suppressing the function and expression of Ascl1 after increasing the expression of Hes1 and Id3 by activating ERK and p38.
(2.51 MB TIF)

Table S1 Single SNP transmission disequilibrium test (TDT) results of stathmin1 SNPs
(0.37 MB TIF)
