# Supplementary figures and images for: Increased Stathmin1 Expression in the Dentate Gyrus of Mice Causes Abnormal Axonal Arborizations
Source: PLoS One. 2010 Jan 6;5(1):e8596. doi: 10.1371/journal.pone.0008596 (PMC2797614; doi:10.1371/journal.pone.0008596)

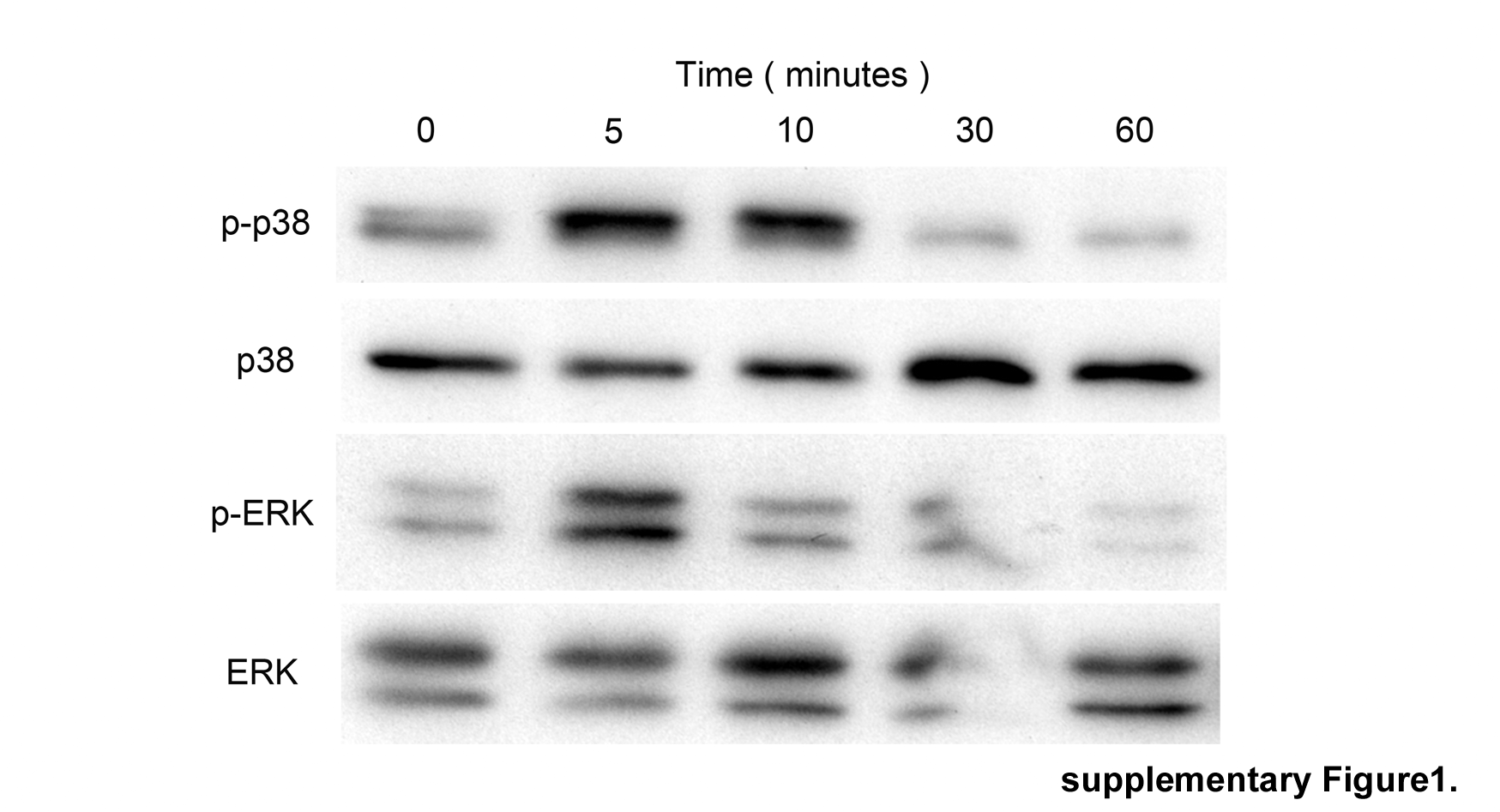

Supplement: Figure S1 — P38 or ERK activation in PC12 cells in response to PACAP stimulation. PC12 cells were treated with 100 nM PACAP. After the stimulation, cells were lysed at each indicated times. Upper 2 panels: Activations of p38 were detected by immunoblotting analysis using the phosphospecific anti-p38 antibody. To control for loading, Western blotting analyses of lysates were performed with anti-p38 antibody. Lower 2 panels: Activations of ERK were also detected by immunoblotting analysis using the phosphospecific anti-ERK antibody. To control for loading, Western blotting analyses of lysates were performed with anti-ERK antibody. Used antibodies as follows; anti-P38 antibody (Cell Signaling. rabbit polyclonal, 1∶1000 dilution), anti-phosopholylated P38 antibody (Cell Signaling. rabbit polyclonal, 1∶1000 dilution), anti-ERK antibody (Cell Signaling. rabbit polyclonal, 1∶1000 dilution), anti-phosopholylated ERK antibody (Cell Signaling. rabbit polyclonal, 1∶1000 dilution). Note: Both P38 and ERK were activated at 5–10 minutes after PACAP stimulation. (0.40 MB TIF) [file pone.0008596.s001.tif]

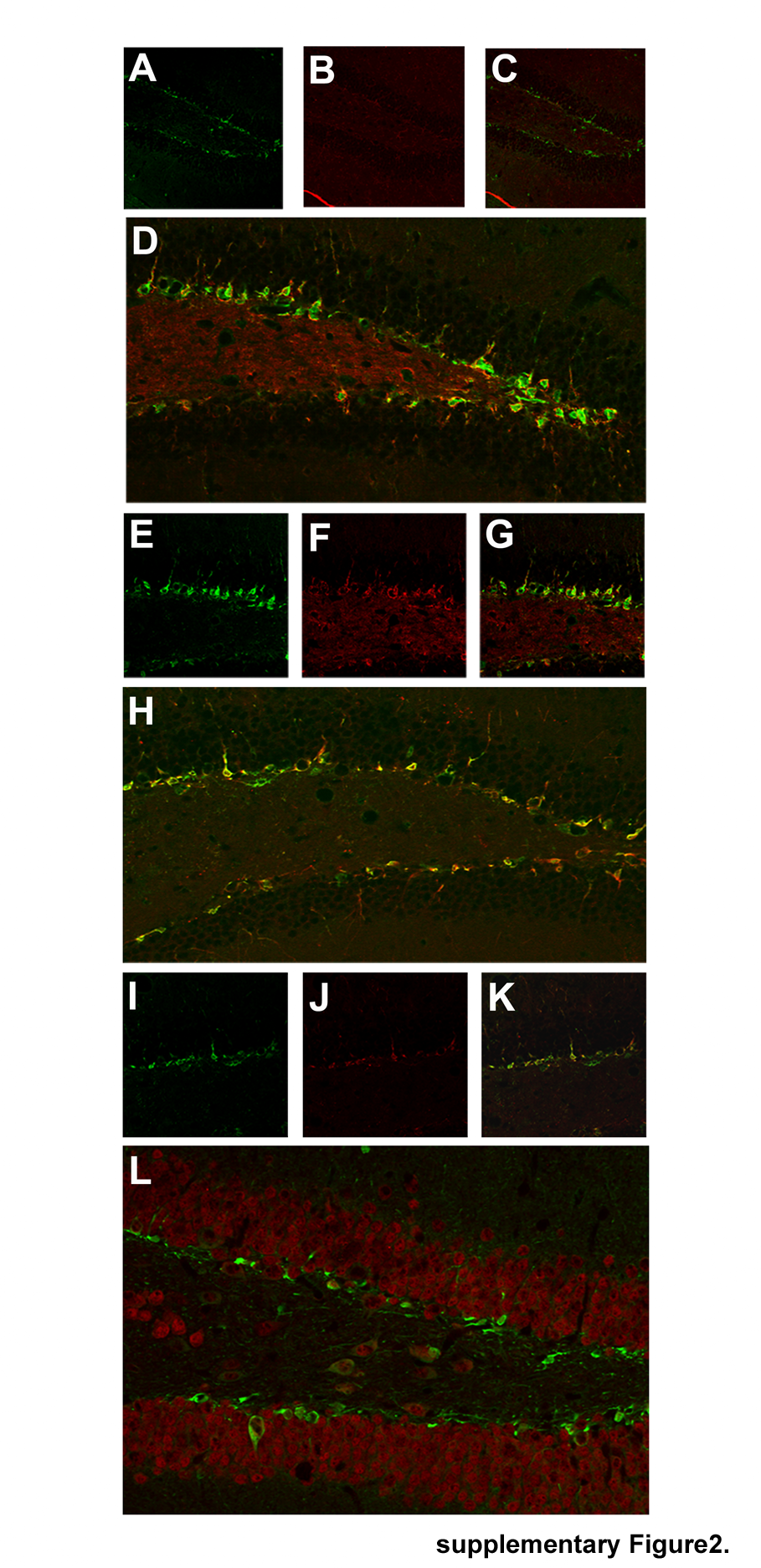

Supplement: Figure S2 — Localisation of stathmin1 with markers of various stages in neurogenesis in dentate gyrus of 8 week-mice. A–C. Mouse hippocampus (dentate gyrus) was stained with anti-stathmin1 and anti-nestin antibodies (A, stathmin1: green, B, nestin: red, C, merged image). D–G. With anti-stathmin1 and anti-PSA-NCAM antibodies (D,E, stathmin1: green, D,F, PSA-NCAM: red, D,G, merged image). E–G: Higher magnification of D. scale bar = 50mm. H–K. With anti-stathmin1 and anti-doublecortin (DCX) antibodies (H,I, stathmin1: green, H,J, DCX: red, H,K, merged image). I–K: Higher magnification of H. scale bar = 50mm. L. Merged image with anti-stathmin1 and anti-NeuN antibodies (stathmin1: green, NeuN: red) scale bar = 50mm. Mice under deep pentobarbital anesthesia were perfused transcardially with 30–50 ml of 4% paraformaldehyde solution. Brain was removed and infused with 30% sucrose overnight at 4oC. After blocking with 5% bovine serum albumin, each sections were incubated overnight at 4 oC with an anti-stathmin1 antibody (GeneTex, Inc. rabbit-polyclonal, 1∶1000 dilution) and either anti-nestin (Becton Dickinson. Mouse-monoclonal, 1∶1000 dilution), anti-PSA-NCAM (abcys. Mouse-polyclonal, 1∶1000 dilution), anti-doublecortin (DCX) (Santa Cruz Bio. Goat-polyclonal, 1∶1000) or anti-NeuN (Chemicon. mouse-monoclonal, 1∶1000 dilution) antibody in 0.01 M PBS containing 0.3% Triton X-100 and 5% BSA. Next, the sections were treated with fluorescent dye (Alexa Fluor 488)-conjugated donkey anti-rabbit IgG (1∶1000 dilution), fluorescent dye (Alexa Fluor 568)-conjugated goat anti-mouse IgG (1∶1000 dilution), and donkey anti-goat IgG (1∶1000) as the secondary antibodies for 1 h at RT in 0.01 M PBS containing 5% BSA. Each sections were observed under a confocal microscope (LSM510, Carl Zeiss). (1.76 MB TIF) [file pone.0008596.s002.tif]

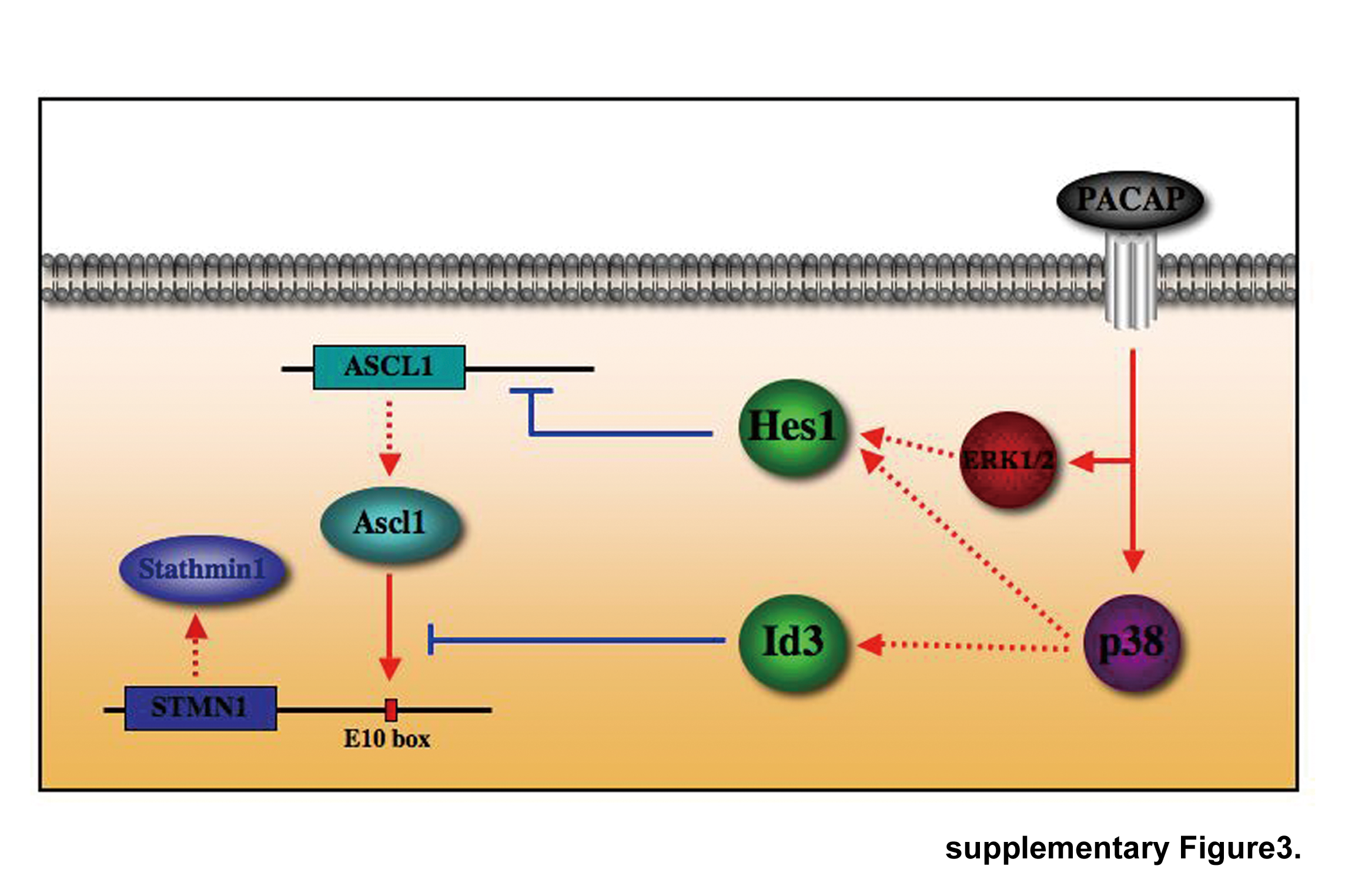

Supplement: Figure S3 — Schematic drawing of the molecular pathway of PACAP regulation on stathmin1 expression shown in this study. A schematic representation of the pathway which PACAP regulates expression of stathmin1 by suppressing the function and expression of Ascl1 after increasing the expression of Hes1 and Id3 by activating ERK and p38. (2.51 MB TIF) [file pone.0008596.s003.tif]

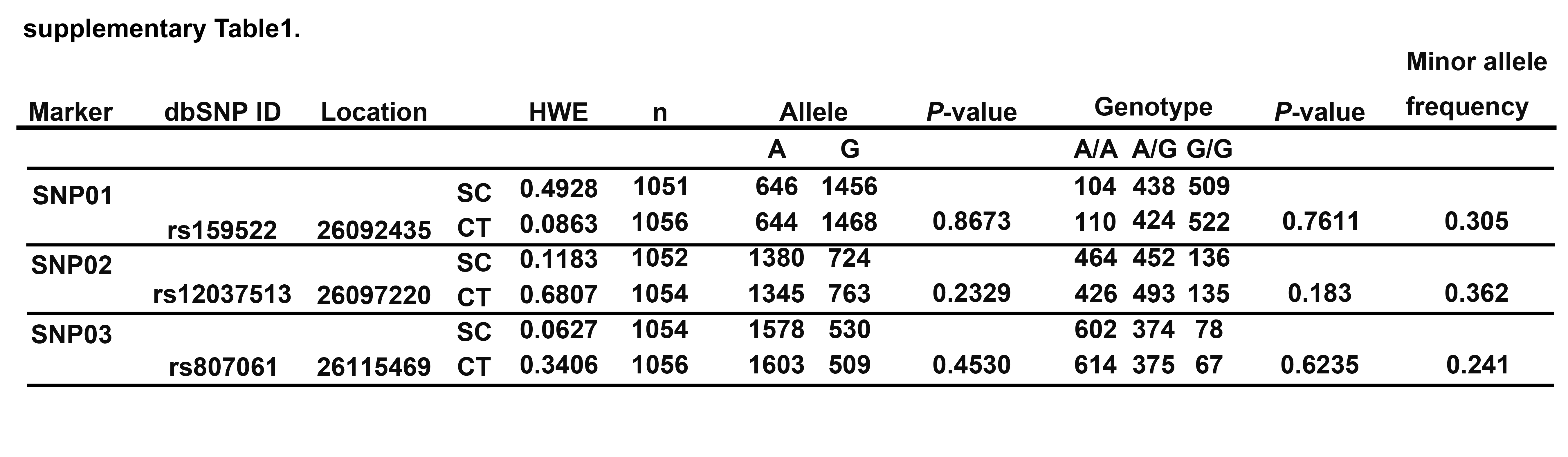

Supplement: Table S1 — Single SNP transmission disequilibrium test (TDT) results of stathmin1 SNPs (0.37 MB TIF) [file pone.0008596.s004.tif]
